# Low-pressure support vs automatic tube compensation during spontaneous breathing trial for weaning

**DOI:** 10.1186/s13613-019-0611-y

**Published:** 2019-12-13

**Authors:** Claude Guérin, Nicolas Terzi, Mehdi Mezidi, Loredana Baboi, Nader Chebib, Hodane Yonis, Laurent Argaud, Leo Heunks, Bruno Louis

**Affiliations:** 1 Médecine-Intensive Réanimation, Hopital Edouard Herriot, CHU de Lyon, Lyon, France; 2 Université de Lyon, Lyon, France; 3 INSERM 955, Créteil, France; 4 CNRS ERL 7000, Créteil, France; 5 Médecine-Intensive Réanimation, CHU de Grenoble-Alpes, La Tronche, France; 6 Université de Grenoble-Alpes, Saint-Martin-d’Hères, France; 7 INSERM 1042, Grenoble, France; 8 Médecine-Intensive Réanimation, Groupement Hospitalier Nord, CHU de Lyon, Lyon, France; 9 Department of Intensive Care, University of Amsterdam, Amsterdam, The Netherlands

**Keywords:** Work of breathing, Respiration, Artificial, Respiratory muscles, Mechanical ventilator weaning, Positive-pressure ventilation

## Abstract

**Background:**

During spontaneous breathing trial, low-pressure support is thought to compensate for endotracheal tube resistance, but it actually should provide overassistance. Automatic tube compensation is an option available in the ventilator to compensate for flow-resistance of endotracheal tube. Its effects on patient effort have been poorly investigated. We aimed to compare the effects of low-pressure support and automatic tube compensation during spontaneous breathing trial on breathing power and lung ventilation distribution.

**Results:**

We performed a randomized crossover study in 20 patients ready to wean. Each patient received both methods for 30 min separated by baseline ventilation: pressure support 0 cmH_2_O and automatic tube compensation 100% in one period and pressure support 7 cmH_2_O without automatic tube compensation in the other period, a 4 cmH_2_O positive end-expiratory pressure being applied in each. Same ventilator brand (Evita XL, Draeger, Germany) was used. Breathing power was assessed from Campbell diagram with esophageal pressure, airway pressure, flow and volume recorded by a data logger. Lung ventilation distribution was assessed by using electrical impedance tomography (Pulmovista, Draeger, Germany). During the last 2 min of low-pressure support and automatic compensation period breathing power and lung ventilation distribution were measured on each breath. Breathing power generated by the patient’s respiratory muscles was 7.2 (4.4–9.6) and 9.7 (5.7–21.9) J/min in low-pressure support and automatic tube compensation periods, respectively (*P* = 0.011). Lung ventilation distribution was not different between the two methods.

**Conclusions:**

We found that ATC was associated with higher breathing power than low PS during SBT without altering the distribution of lung ventilation.

## Background

Weaning intensive care unit (ICU) patients from invasive mechanical ventilation relies on a daily screening for eligibility, and if present, a spontaneous breathing trial (SBT) testing patient capacity to breathe without respiratory assistance [1]. SBT can be done by either setting a low-pressure support ventilation (PS) level or allowing the patient to breathe spontaneously through the endotracheal tube without any support from the ventilator (T-piece). Even though both SBT methods are almost equivalent to predict weaning outcome [2] [3], American guidelines suggested performing the first weaning trial using PS rather than T-piece to hasten extubation [4]. Recent data are consistent with this recommendation [5].

Low PS level was thought as a mean to compensate for the endotracheal tube airflow resistance (*R*_ET_) [6]. However, the fact that low PS actually reduced work of breathing (WOB) as compared to T-piece [7] questioned this concept. The WOB after extubation, which was similar as that during SBT [8, 9], was better predicted with T-piece than with low PS [10]. A meta-analysis of studies comparing at least two SBT techniques found that low PS reduced WOB by 30% as compared to T-piece [10]. Therefore, low PS should provide a further respiratory assistance beyond *R*_ET_ compensation. We reasoned that the only way to provide a support that would just compensate for *R*_ET_ is the automatic tube compensation (ATC) option available in many ICU ventilators [11]. ATC works as a closed-loop during insufflation (and/or exsufflation) to compensate for the non-linear pressure–flow relationship across endotracheal tube or tracheostomy canula [12].

Therefore, in patients ready to wean from mechanical ventilation we aimed at comparing both techniques used in clinical practice, namely low PS/PEEP and ATC mode with PEEP [13]. Our hypothesis was that the WOB is greater in ATC than in the low PS/PEEP, and, if it is true, low PS provides support beyond *R*_ET_ compensation. Assisted spontaneous breathing is increasingly suspected to damage the lung by promoting excessive trans-pulmonary pressure in the most ventral lung regions [14]. Therefore, our secondary objective was lung ventilation distribution, with the hypothesis of a more ventral distribution with low PS than ATC at same PEEP.

## Methods

The protocol was approved by an Ethics Committee and the National Agency for drug safety in France and registered (NCT02939963 in clinical.trial.gov).

### Patients

Patients were eligible if they met all the following inclusion criteria: (1) age ≥ 18 years; (2) intubated and mechanically ventilated for acute respiratory failure for at least 24 consecutive hours; (3) able to tolerate PS 10–15 cmH_2_O with total respiratory rate 25–35 breaths/min and expired tidal volume 6–8 ml/kg predicted body weight; (4) meeting criteria for SBT (see Additional file 1); (5) under EVITA XL ICU ventilator (Dräger, Germany); (6) agreement to participate from the patient or her/his next of kin. The list of non-inclusion criteria is provided in Additional file 1.

### Measurement set-up

We measured airflow by using a linear pneumotachograph (3700 series, Hans Rudolph, Shawnee, Kansas) and airway pressure (Paw) at the proximal tip of the endotracheal tube. We measured esophageal pressure (Pes) by using a 5Fr specific catheter balloon (CooperSurgical, Inc., Trumbull, CT) descended down to the lower third of esophagus through the nostril. We assessed the proper position of the Pes device [15] and the amount of nonstressed air volume into the esophageal balloon [16]. Paw and Pes were connected to pressure transducers (Gabarith PMSET 1DT-XX, Becton-Dickinson, Singapore). Pressure transducers and pneumotachograph were calibrated using a manometer (717 1G, Fluke Biomedical, Everett, Washington) and a precision rotameter (Houdec Glass, Martin Medical, Lyon, France), respectively, at room temperature in each experiment. The set-up had a flow-resistance of 0.79 cmH_2_O/l/s and a dead space of 20 ml. During the experiment the heated-humidifier was working on and the pneumotachograph was not warmed to avoid any risk of endotracheal tube obstruction.

We wrapped the thorax at the 5–6th intercostal space with a 16-electrode electrical impedance tomography (EIT) belt. The belt was connected to an EIT monitor (Pulmovista 500, Dräger, Lubeck, Germany). EIT device measured changes in impedance across the thorax from the measurement of surface potential differences resulting from the application of a low-intensity alternate electrical current generated by pairs of electrodes and rotating around the thorax at a rate of 20 Hz.

### Protocol

This was a crossover study with two treatment arms. Each included patient received both arms in a computer-generated random order. In the ATC arm, the ventilator was set at PS 0 cmH_2_O, with the shortest rising time, cycling-off 25% of maximal inspiratory flow, PEEP 4 cmH_2_O and ATC on with 100% inspiratory compensation for the patient’s endotracheal tube size. Expiratory ATC was not activated because it was not available in the ventilators used in present study. In the low-PS arm, settings were the same except for 7 cmH_2_O PS and ATC off. Each treatment period was applied during 30 min and was separated by a 30-min period during which baseline ventilator settings were resumed. If the patient did not tolerate SBT (see Additional file 1 for criteria) he/she was switched back to the baseline ventilator settings and qualified as SBT failure.

During the last 2 min of each treatment period, Paw, Pes and airflow analog signals were continuously recorded at 200 Hz by using a data logger (Biopac MP150, Biopac, Inc., Goleta, CA). We obtained Paw at 100 ms (P0.1) by activating a specific function built into the ventilator. Five brief end-expiratory occlusions were automatically generated by the ventilator after manually pushing on a specific button at random during the 2 min of the recording. In the same time, EIT signals were continuously recorded. Paw, Pes, airflow signals were stored for off-line analysis by using Acqknowledge 4.0 version (Biopac, Inc., Goletta, CA). The same was done for the EIT signals by using a specific software (EITDataAnalysisTool 6.1, Dräger, Lubeck, Germany).

### Data analysis

**Fig. 1:**
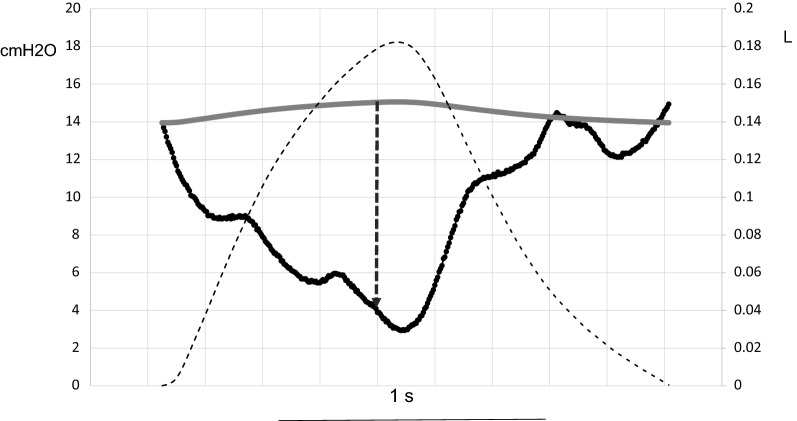
Method to compute muscular pressure (Pmus) over one breath. Scope mode displays over time the tidal volume (black dashed line), the recorded esophageal pressure (Pes,dyn, black continuous line) and the computed static esophageal pressure (Pes,st gray continuous line) (record from patient #16 in the automatic tube compensation group). At any time Pmus is the distance (black dashed vertical arrow) pertaining to Pes,dyn–Pes,st. Scale in cmH_2_O in the left Y axis and in liter (l) in the right Y axis. Time scale (1 s) is indicated

Over each recorded breath the measurements were automatically performed under an in-house software developed with the Matlab scripting language. Tidal volume (VT) and respiratory rate were obtained from the flow signal. Inspiration was defined as the flow crossing zero. Resistive (*R*) and elastic (*E*) components of WOB done by the patient were obtained from the Campbell diagram and the total WOB was the sum of the *R* and *E* components. Muscular pressure (Pmus) was computed as the difference between Pes and VT times chest wall elastance in each breath (Fig. 1). Chest wall elastance was computed as the change in Pes over the breath divided by 4% vital capacity expected for gender, age and height [17]. The *R* and *E* components of breathing power were the product of each WOB component to respiratory rate and expressed as J/min. The total breathing power was the sum of its *R* and *E* components. The pressure–time product of inspiratory muscles (PTPmus) was the area of Pmus over the inspiration in each breath multiplied by the respiratory rate. Intrinsic PEEP was measured as the Pes deflection from the onset of inspiratory effort to the first zero flow. No correction was made for gastric pressure.

P0.1 was measured on the Paw tracings recorded in the data logger at 100 ms after the first zero flow. The values of the 5 measurements per condition were averaged.

We assessed the functioning of the ventilator in each mode by measuring PEEP, maximal deflection in Paw at the time of inspiratory effort (DPtrig), time delay between onset of inspiratory effort to return to baseline PEEP (DTtrig), maximal inspiratory pressure and maximal inspiratory flow (see Additional file 1).

The EIT signals were processed with the EIT and diffuse optical tomography reconstruction software (EIDORS) [18] licensed under the GNU general public (http://eidors3d.sourceforge.net/) associated with the Matlab scripting language (see Additional file 1).

The pressure drop across the endotracheal tube was evaluated from general mechanical law as described [19]. This pressure drop is a function of the instantaneous flow rate and the endotracheal tube geometry (length and diameter). We measured the pressure generated by the ventilator and we computed the ideal pressure that would only be needed to compensate for RET. The difference between this ideal pressure and effective Paw was computed by summing the instantaneous difference (see Additional file 1 for more details).

### Statistical analysis

The primary end-point was the total breathing power generated by the patient’s respiratory muscles and was used to power the study. We set total breathing power to 10 J/min in the low-PS arm reference group [7], and a 4 J/min clinically relevant increase in total breathing power with ATC with a 4 J/min standard deviation [7]. At first and second risk orders of 5% and 20%, respectively, 16 patients were needed (Epi-Info software). Assuming a 10% rate of patients with missing data a total of 20 patients should be enrolled in the study.

The values were expressed as median (1st–3rd quartiles) and compared by non-parametric Wilcoxon signed rank test between the two arms. For the primary end-point, we furthermore tested the period effect and the treatment–period interaction [20]. If the period has a significant effect the analysis would be adjusted for the period. If the interaction between treatment and period is significant only the first period will be used. Correlation between variables was assessed by using Spearman rank correlation. The statistical analysis was performed by using the R software 3.5.2 version [21]. *P* value < 0.05 was taken as the statistical significant threshold. No correction for multiple comparisons was done.

## Results

### Patients sample description

**Fig. 2:**
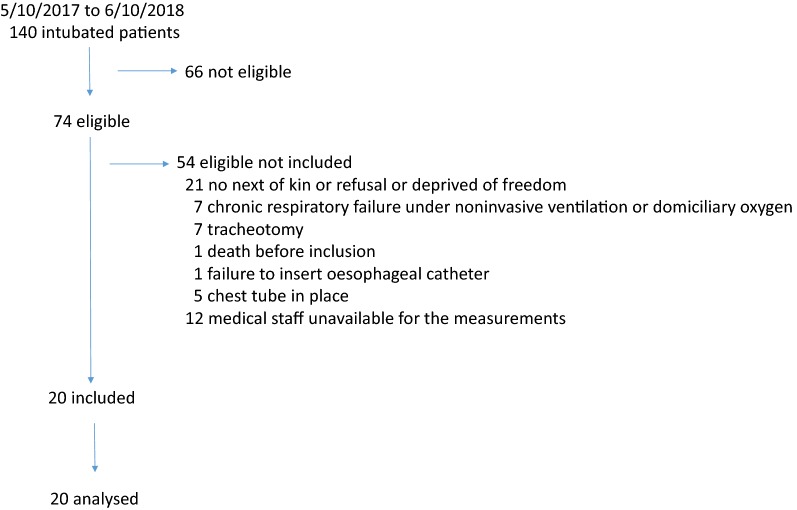
Flowchart of the patients during the study period

Twenty patients were included between August 23, 2017 and October 5, 2018 (Fig. 2 and Table 1). All but two were admitted in a medical setting. Time from intubation for acute respiratory failure to study was 7 (4–14) days. The main cause of acute respiratory failure requiring intubation was: community-acquired pneumonia (*n* = 9), aspiration (*n* = 2), fluid overload (*n* = 3), and pulmonary embolism, pleural space infection, acute respiratory distress syndrome of unknown origin, hospital-acquired pneumonia, coma, and chronic obstructive pulmonary disease (*n* = 1 for each). SBT under present investigation was the first for 16 patients, the second for 2 and the third for 2. All but one succeeded the SBT and were extubated right away thereafter. No adverse event was observed during the study. The day of the study PaO_2_/F_I_O_2_ was 287 (206–362) mmHg, PaCO_2_ 38 (35–41) mmHg, pH 7.45 (7.42–7.49). EIT data were lacking in three patients due to technical problem with the belt functioning. The baseline breathing pattern, breathing power and inspiratory effort at the ventilatory settings shown in Table 1 were the following: VT 0.38 (0.31–0.48) l, respiratory rate 26 (23–31) breaths/min, minute ventilation 9.6 (8.4–11.4) l/min, DTtrig 0.17 (0.14–0.20) s, DPtrig − 1.9 (− 2.6; − 1.4) cmH_2_O, maximal Paw 14 (12–16) cmH_2_O, and total breathing power 8.6 (3.3–13.3) J/min and 3.8 (1.6–7.2) and 4.0 (1.5–5.8) J/min for its resistive and elastic components, respectively.

**Table 1:** Data entry of 20 patients the day of the study comparing automatic tube compensation and low-pressure support during spontaneous breathing trial

Patient	Age (years)	Gender	BMI (kg/m^2^)	SAPSII	SOFA	ETT ID (mm)	PS (cmH_2_O)	VT (ml/kg PBW)	PEEP (cmH_2_O)	Status at ICU discharge
1	64	F	20	67	2	7.0	3	10	4	D
2	60	F	30	31	3	7.0	10	9	5	A
3	64	M	30	35	5	7.5	7	10	4	A
4	55	F	13	34	4	7.0	7	6	5	A
5	59	M	23	31	13	8.0	10	7	4	A
6	70	M	29	63	9	8.0	7	7	5	A
7	62	M	36	43	4	8.0	12	4	5	A
8	62	F	23	52	13	7.5	10	0	5	A
9	78	M	30	67	8	7.5	10	6	4	A
10	71	M	25	53	7	8.0	12	12	5	A
11	79	M	28	50	7	8.0	7	6	5	A
12	64	M	31	84	8	7.5	7	8	4	A
13	47	M	23	46	4	7.5	7	8	5	A
14	68	M	28	66		7.5	8	6	5	A
15	40	F	29	40	3	7.5	7	7	4	A
16	59	M	25	59	3	7.5	10	5	5	A
17	60	M	42	35	12	8.5	10	6	5	A
18	66	M	24	57	9	8.5	10	8	5	A
19	19	M	33	37	2	8.0	10	4	5	A
20	63	M	22	67	10	8.5	10	5	5	D
Median (1st–3rd quartiles)	63 (59–67)	15 M 5 F	28 (23–30)	51 (37–64)	7 (4–9)		10 (7–10)	7 (6–8)	5 (4–5)	

*BMI* body mass index, *SAPS* simplified acute physiology score, *SOFA* sequential organ failure assessment, *ETT* endotracheal tube, *ID* internal diameter, *PS* pressure support, *VT* tidal volume, *PBW* predicted body weight, *PEEP* positive end-expiratory pressure, *ICU* intensive care unit, *M* male, *F* female, *D* dead, *A* alive

### Effects of SBT mode on breathing pattern and inspiratory effort

VT, inspiratory and expiratory times were not different between the two periods (Table 2). Respiratory rate was 27 (21–33) vs. 25 (21–28) breaths/min (*P* = 0.007), and minute ventilation 9.5 (7.4–11.7) vs. 9.4 (8.5–10.1) (*P* = 0.22), in ATC and PS periods, respectively.

**Table 2:** Breathing pattern and ventilator functioning in automatic tube compensation and low-pressure support periods during spontaneous breathing trial

	ATC	Low PS	*P* value
Tidal volume (l)	0.35 (0.30; 0.45)	0.35 (0.32; 0.50)	0.22
Tidal volume (ml/kg predicted body weight)	5.3 (4.5; 6.3)	5.6 (4.7; 7.7)	0.21
Respiratory rate (breaths/min)	27 (21; 33)	25 (21; 28)	0.007
Minute ventilation (l/min)	9.5 (7.4; 11.7)	9.4 (8.3; 10.4)	0.22
Respiratory rate/tidal volume ratio (breaths/min/l)	77 (52; 105)	70 (43; 88)	0.048
Inspiratory time (s)	0.95 (0.78; 1.11)	0.88 (0.80; 1.05)	0.79
Expiratory time (s)	1.44 (1.10; 1.78)	1.72 (1.36; 1.96)	0.05
Inspiratory/expiratory time ratio (%)	0.67 (0.54; 0.81)	0.55 (0.49; 0.71)	0.04
PEEP (cmH_2_O)	5 (4; 5)	5 (4–5)	0.60
DPtrig (cmH_2_O)	− 2 (− 3; − 1)	− 2 (− 3; − 2)	0.60
DTtrig (s)	0.19 (0.12; 0.22)	0.15 (0.13; 0.19)	0.007
Maximal inspiratory pressure (cmH_2_O)	8 (7; 8)	12 (12–12)	0.00006
Maximal inspiratory flow (l/s)	1 (1; 1)	1 (1; 1)	0.18

*ATC* automatic tube compensation, *PS* pressure support, *PEEP* positive end-expiratory pressure, *DPtrig* maximal depression in airway pressure from PEEP during activation of inspiratory trigger, *DTtrig* time delay between onset of inspiratory effort and return to baseline PEEP before inspiratory valve opening

Values are median (1st; 3rd quartiles)

For the total breathing power generated by the patient’s respiratory muscles, the primary end-point of the study, there were no statistically significant effect of neither the period nor the interaction between period and treatment (Table 3). It was significantly higher with ATC [9.7 (5.7–21.9)] than with low PS [7.2 (4.4–9.6)] J/min (*P* = 0.011). The same was true for its resistive (*P* = 0.035) and elastic components (*P* = 0.0096) (Table 3). Between ATC and low PS, PTPmus was 232 (181–349) and 153 (120–251) cmH_2_O s/min (*P* = 0.0009).

**Table 3:** Results of the cross-over design analysis for the breathing power (the primary end-point of the study) in the automatic tube compensation and the low-pressure support periods during spontaneous breathing trial in 20 patients

	ATC	Low PS	Treatment effect	Period effect	Treatment × period interaction (carry over effect)
Total breathing power (J/min)	9.7 (5.7–21.9)	7.2 (4.4–9.6)	0.011	0.060	0.175
Resistive breathing power (J/min)	4.8 (1.6–9.9)	3.1 (1.9–4.6)	0.035	0.086	0.116
Elastic breathing power (J/min)	5.0 (3.6–7.7)	3.5 (2.4–5.2)	0.0096	0.081	0.238

Values are median (1st–3rd quartiles)

*ATC* automatic tube compensation, *PS* pressure support, *J/min* joules per minute

P0.1 was 3.3 (1.6–4.6) cmH_2_O in ATC and 1.9 (1.4–3.3) cmH_2_O in low-PS group (*P* = 0.03).

**Fig. 3:**
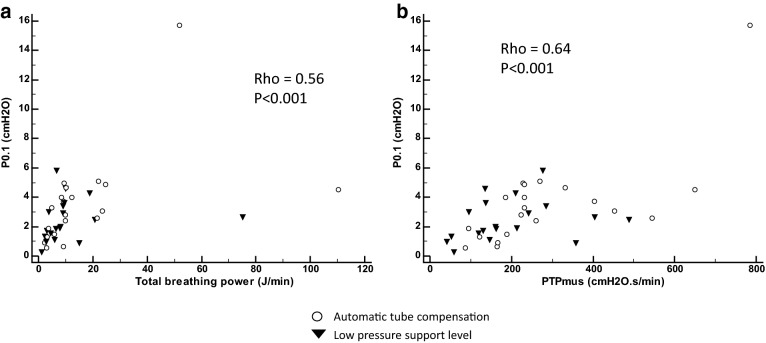
Relationships between airway pressure at 100 ms after occlusion (P0.1) and breathing power (**a**) and pressure time product performed by the respiratory muscles (PTPmus) (**b**). Rho Spearman correlations are shown for all over the data points. Open circles for automatic tube compensation group and black triangles for low-pressure support group

There was a significant linear relationship between P0.1 and both total breathing power and PTPmus over all the data points (Fig. 3).

### Ventilator support functioning in SBT

Between ATC and low PS the maximal Paw was, as expected, greater with low PS than with ATC (Table 2). It is worth mentioning that ATC generated a roughly 3 cmH_2_O pressure assistance above PEEP. DTtrig was longer with ATC than with low PS by 40 ms roughly, but this value is likely not clinically relevant even though it reached statistical significance (Table 2).

The pressure generated by the ventilator surpassed the ideal pressure by 25% in ATC and by 400% in low-PS mode. That means that the ventilator is further away from the ideal pressure with low PS than with ATC.

### Electrical impedance tomography

At baseline, global inhomogeneity index was 0.56 (0.43–0.67), center of ventilation 53 (46–58), anterior-to-posterior tidal impedance distribution 0.7 (0.53–1.84) and coefficient of variation of ventilation 0.91 (0.81–0.99). EIT indexes were not different between ATC and low-PS periods (Table 4). The Spearman’s coefficient of rank correlation values between EIT indexes and breathing power performed over all data points are provided in Table 5.

**Table 4:** Electrical impedance tomography indexes in automatic tube compensation and low-pressure support periods during spontaneous breathing trial in 17 patients

	ATC	Low PS	*P* value
Anterior-to-posterior impedance ratio	0.69 (0.58–1.59)	0.86 (0.49–1.61)	0.55
Center of ventilation	54.8 (46.3–58.1)	53.7 (46.0–60.2)	0.82
Global inhomogeneity index	0.62 (0.47–0.80)	0.65 (0.50–0.75)	0.35
Coefficient of variation	0.95 (0.81–1.82)	0.96 (0.77–1.39)	0.68

Values are median (1st–3rd quartiles)

*ATC* automatic tube compensation, *PS* pressure support

**Table 5:** Spearman’s coefficient of rank correlation between electrical impedance tomography indexes and breathing power performed over all the data points

Breathing power	Electrical impedance tomography indexes			
	Anterior-to-posterior ratio	Center of ventilation	Coefficient of variation	Global inhomogeneity index
Elastic	− 0.38*	0.39*	− 0.18	0.34*
Resistive	− 0.39*	0.41*	− 0.18	0.43*
Total	− 0.36*	0.37*	− 0.16	0.41*

* *P* < 0.05

## Discussion

The main finding of present study was that breathing power was significantly higher with ATC than with low PS and without ATC at same PEEP. This result confirms our working hypothesis that low PS provides respiratory assistance above *R*_ET_.

### Work of breathing

Similar result was reported by Kuhlen et al. [22], who compared PS 7 cmH_2_O and PEEP 7 cmH_2_O, ATC and PEEP 8 cmH_2_O and T-piece trial and PEEP 0 cmH_2_O in 12 patients undergoing SBT. It is worth mentioning that the investigators used a previous ventilator (Evita 4 vs. Evita XL in present study), endotracheal tubes much larger than in present study, the amount of *R*_ET_ compensation was not indicated, and distribution of ventilation not assessed. Therefore, present study extended previous results [22]. Interestingly, the values of PTPmus and P0.1 were very close between each study in both periods. Also remarkable is that the same respiratory assistance level, amounting to 3 cmH_2_O, was observed with the ATC mode in both studies. The previous generation of Evita ventilators is very close to the next one in the amount of support generated in ATC mode. Taking these two studies together, it can be concluded that low PS of 7 cmH_2_O does not only compensate for *R*_ET_, but also provides a real respiratory assistance and unloads the patient as compared to a spontaneous breathing mode that compensates only for *R*_ET_. Whether or not other brands of ventilators would result in same respiratory breathing assistance in ATC remains to be investigated.

Cabello et al. compared in 14 difficult-to-wean patients in a cross-over design PS 7 cmH_2_O PEEP 5 or 0 cmH_2_O and T-piece for 60 min each [7]. From the former to the latter SBT method WOB and PTPmus increased progressively. Of notice breathing power was of same magnitude in ATC group of present study and low-PS PEEP 0 cmH_2_O in [7].

We found a significant correlation between P0.1 and breathing power. Therefore, P0.1 could be a tool to titrate the level of PS during SBT.

### Regional distribution of ventilation

Assessing distribution of ventilation at the time of weaning has been poorly investigated. Such assessment is, however, important because assisted spontaneous breathing may promote overdistension in either ventral or dorsal lung regions [23, 24]. Therefore, lung protection should be a target as important at the time of weaning as in earlier stage of mechanical ventilation [25]. On the other hand, loss of aeration may occur during SBT and has been shown to predict weaning outcome when assessed by lung ultrasound [26].

We used EIT, which is a radiation-free, non-invasive method to determine regional ventilation distribution [27]. It has a favorable comparison with gold standard technique to measure lung ventilation quantitatively [28]. In present study, the EIT indexes were similar between each ventilator mode. There is no previous study for ATC and low PS on EIT indexes during SBT to compare with. Mauri et al. compared two PS levels at same PEEP of 7 cmH_2_O in 10 ARDS patients [29]. Dorsal distribution of lung ventilation increased and distribution of ventilation was more homogeneous in low (3 cmH_2_O) as compared to high (12 cmH_2_O) PS levels [30]. In present study the difference in PS level between low PS and ATC averaged 4 cmH_2_O (Table 3) and may be not sufficient to induce changes in ventilation distribution. Moreover, our patients were ready to wean, and we can expect that their potential lung inhomogeneity is less marked than in ARDS patients. However, present EIT findings suggest that ATC would not increase lung ventilation heterogeneity as compared to low PS and low PEEP that is likely to maintain lung ventilation homogeneous. We found that anterior-to-posterior lung ventilation distribution, center of ventilation and global inhomogeneity EIT indexes correlated with breathing power, even though the correlation was weak. These findings would suggest that heterogeneity of ventilation would increase with breathing power setting the risk of lung injury [31]. Hsu et al. compared assisted volume-controlled mechanical ventilation to ATC 100% PEEP 5 cmH_2_O in 16 patients under invasive mechanical ventilation for 40 days [32]. The distribution of ventilation was higher in dorsal than in ventral regions with ATC. Zhao et al. found that greater weaning success in patients with better redistribution towards the dorsal lung regions [33]. Bickenbach et al. found that EIT indexes, like impedance ratio between dorsal and ventral lung regions, intra-tidal variation of impedance, global inhomogeneity index, regional ventilation delay, end-expiratory lung impedance, may predict the success or failure of SBT [34]. We found meaningless in present study to compare the single patient who failed with the 19 who succeeded SBT.

### Limitations and strengths

Present study was limited by the lack of a T-piece without ATC condition and of WOB measurement after extubation. This choice was made because our study was hypothesis-driven. We did not measure gastric pressure to take into account expiratory muscles contraction. However, we did not observe any significant Pes change during expiration. Our study has strengths as it is the first assessing the distribution of lung ventilation in ATC as compared to low PS during SBT. The sample size was a priori computed.

### Clinical implications

Present study was crossed-over and not designed to determine whether one mode would be better than the other to predict SBT failure, or to be used during the whole weaning process. Cohen et al. [35] performed a randomized controlled trial in ready-to-wean patients. They compared ATC 100% to 7 cmH_2_O PS both at same PEEP of 5 cmH_2_O for 1 h to predict extubation outcome. The rate of successful extubation was the same between the two periods. This study was close to the present one regarding SBT strategies. Of notice COPD patients accounted for 8 and 11% in the ATC and low-PS periods, respectively. Applying our results on breathing power to the study by Cohen et al. [35] would suggest that even low PS can reduce significantly WOB it would not be better than ATC for SBT outcome. A recent large randomized controlled trial compared 578 patients who received a 2-h T-piece trial to 575 patients who received low PS (8 cmH_2_O) for 30 min without PEEP in each [5]. The primary outcome was the rate of successful extubation. It was 82.3% in the low-PS group and 74% in the T-piece group (*P* = 0.001). Whereas T-piece trial could be the best test to replicate the physiological conditions after extubation, PS trial seems to be the best test to hasten extubation without an increased risk of reintubation. ATC would deserve a trial during weaning because it shares both advantages of low PS by providing some ventilatory assistance and of SBT in replicating physiological conditions increased. We can also note that poor trigger performance (DTtrig > 150 ms) of the ventilator would make it more difficult to see difference between low PS, ATC or SBT. It is worth mentioning that SBT success is not the same as weaning success, which is commonly assessed 48 h after extubation [1]. Furthermore, recent data indicate that clinicians do not offer a SBT in almost 50% of patients and that successful SBT is not always followed by extubation [36]. The data of the large prospective epidemiological study (Wean safe) are coming up soon and would tell us more about the practice of weaning all over the world (NCT03255109).

## Conclusions

We found that ATC was associated with higher breathing power than low PS during SBT without altering the distribution of lung ventilation.

## Supplementary information

**Additional file 1.** Methods.

## Data Availability

All data generated or analyzed during this study are included in this published article [and its additional information files].

## Abbreviations

ATC: automatic tube compensation
DPtrig: maximal airway pressure required to open the inspiratory valve
DTtrig: time required to generate DPtrig
*E*: elastance
EIT: electrical impedance tomography
ICU: intensive care unit
P0.1: airway pressure 100 ms after onset of inspiratory effort
Paw: airway pressure
PEEP: positive end-expiratory pressure
Pes: esophageal pressure
Pmus: muscular pressure
PS: pressure support
PTPmus: pressure time product of respiratory muscles
*R*: resistance
*R*_ET_: resistance of endotracheal tube
SBT: spontaneous breathing trial
VT: tidal volume
WOB: work of breathing
