# Supplementary material for: Low-pressure support vs automatic tube compensation during spontaneous breathing trial for weaning
Source: Ann Intensive Care. 2019 Dec 13;9:137. doi: 10.1186/s13613-019-0611-y (PMC6911134; doi:10.1186/s13613-019-0611-y)
Supplement: Supplementary file 1 — Additional file 1. Methods. [file 13613_2019_611_MOESM1_ESM.docx]

PATIENTS AND METHODS

Patients

Patients were eligible if they met all the following inclusion criteria: 1) age 18 years or more;

2) intubated and mechanically ventilated for at least 24 consecutive hours; 3) able to tolerate PS 10-15 cmH2O with total respiratory rate 25-35 breaths/min and expired tidal volume 6-8 ml/kg predicted body weight; 4) meeting criteria for SBT: able to answer simple questions, no ongoing intravenous sedation, norepinephrine ≤ 1 mg/h, dobutamine ≤ 20 mg/h, fraction of inspired oxygen (FIO2 ≤ 50%), PEEP ≤ 5 cmH2O, respiratory rate ≤ 35 breaths/min, trans-cutaneous oxygen saturation (SpO2) ≥88%); 5) under EVITA XL or V500 ICU ventilator (Dräger, Germany); 6) agreement to participate from the patient or her/his next of kin.

Patients were not included if any of the following criteria was present: 1) chronic respiratory failure under long term home oxygen therapy and/or noninvasive ventilation before ICU admission; 2) tracheotomy; 3) nasogastric tube contra-indicated; 4) thoracic tube in place;5) no agreement to participate; 5) under justice protection; 6) deprived of freedom; 7) pregnant or breastfeeding; 8) not affiliated to social insurance; 9) involved into another study that may interfere with present study.

Methods

Each included patient received in a cross over design two treatment arms in a computer-generated random order. In the ATC arm, the patient breathed spontaneously through the endotracheal tube connected to the ventilator set at PS 0 cmH_2_O, PEEP 4 cmH_2_O and ATC on with 100% inspiratory compensation for the patient’s endotracheal tube size. Expiratory ATC was not available. In the low PS arm, patients received 7 cmH_2_O PS, PEEP 4 cmH_2_O, ATC off. Each treatment period was applied during 30 minutes and was separated by a 30-minute period of baseline ventilator settings. If any of the followings happened during the 30-minute treatment period, the patient was switched back to the baseline ventilator settings and qualified as SBT failure: respiratory rate > 35 breaths/min, neck accessory muscles contraction, agitation, sweats, mottled skin, systolic arterial pressure greater than 160 mmHg or lower than 90 mmHg for more than 5 minutes, heart rate greater than 140/min or lower than 50/min for more than 5 minutes, thoracic pain, SpO2<88% for more than 5 minutes, impairment of conscious level.

The EIT signals were processed with the EIT and diffuse optical tomography reconstruction software (EIDORS) (2) licensed under the GNU general public (<http://eidors3d.sourceforge.net/>) associated with the Matlab scripting language. In this reconstruction we used the “adult male 16el lungs” GREIT model (3). Once the sequence of raw EIT images were built (32x32 pixels, 30 images/s), the EIT waveform (global impedance change as a function of time) was generated by summing the value of all pixels in each image. The local maxima and minima of this waveform are used to separate cycles in order to infer a functional image representing for each pixel the mean tidal volume. Then, from this functional image, the following EIT indexes were measured: global inhomogeneity index, center of ventilation, anterior-to-posterior tidal impedance distribution and coefficient of variation of ventilation (19). Change in EIT impedance was expressed as arbitrary units.

Ideal pressure

To infer the ideal pressure (IDP) that the ventilator should generate to exactly compensate RET we used the works of Lofaso *et al.* (1) showing that the pressure drop across an adult endotracheal tube follows the Blasius formula. So, the ideal pressure is given by:

$$\text{IDP}\text{(t) }\text{=}\text{ }\text{PEEP}\text{ }\text{+}\overset{Blasius formula}{\overbrace{\text{0.2411}\frac{\rho\cdot\nu^{0.25}\cdot L_{ETT}\cdot{\dot{V}(t)}^{1.75}}{d^{4.75}}}}\text{ }if Rey=\frac{4\cdot\dot{V}(t)}{\pi\cdot d\cdot\nu}\text{≥ }\text{2400 }$$

Where ρ is the density of air, n is the cinematic viscosity of air, L_ETT_ the length of the endotracheal tube, d is its internal diameter and $\dot{V}$ the flow rate. For the low value of the flow rate, i.e., when the Reynolds number (Rey) was less than 2400 in the endotracheal tube, we used the classical Hagen–Poiseuille equation for the pressure drop across the ETT.

$$\text{IDP}\text{(t) }\text{=}\text{ }\text{PEEP}\text{ }\text{+}\text{ }\overset{Hagen-Poiseuille}{\overbrace{\text{128}\frac{\rho\cdot\nu\cdot L_{ETT}\cdot\dot{V}(t)}{\pi{\cdot d}^{4}}}} if Rey\text{ < }\text{2400 }$$

IDP depends on the flow rate and on the endotracheal tube dimensions. In order to compare the ability of the 2 mode (ATC and low PS) to generate a pressure allowing to exactly compensate RET we measured:

$$I\_err=\frac{\int_{t_{startinspiration}}^{t_{endinspiration}} abs(P_{aw}-IDP)\cdot\partial t}{\int_{t_{startinspiration}}^{t_{endinspiration}} (IDP-PEEP)\cdot\partial t}$$

See example in the figure O1 below:

Figure O1: *I_err* is the ratio between the area of the pink-blue pattern and the area of the white-blue pattern. This index is the absolute difference between the pressure generated by the ventilator and IDP normalized by the value of IDP.

The figure O2 shows the methods to determine the maximal deflection in airway pressure at the time of inspiratory effort (DPtrig) and the time delay between onset of inspiratory effort to return to baseline PEEP (DTtrig).


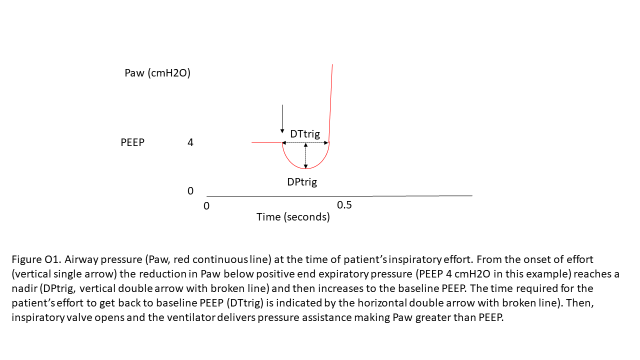


REFERENCES

1. Lofaso F, Louis B, Brochard L, Harf A, Isabey D. Use of the blasius resistance formula to estimate the effective diameter of endotracheal tubes. *Am Rev Respir Dis* 1992;146:974-979.

2. Adler A, Lionheart WR. Uses and abuses of eidors: An extensible software base for eit. Physiol Meas 2006;27:S25-42.

3. Adler A, Arnold JH, Bayford R, Borsic A, Brown B, Dixon P, Faes TJ, Frerichs I, Gagnon H, Garber Y, et al. Greit: A unified approach to 2d linear eit reconstruction of lung images. Physiol Meas 2009;30:S35-55.
